# Incidence of contrast-induced neurotoxicity following endovascular treatment of unruptured intracranial aneurysms: a single-centre cohort study

**DOI:** 10.1007/s13760-024-02643-5

**Published:** 2024-09-26

**Authors:** Frederick P. Mariajoseph, Leon T. Lai, Justin Moore, Ronil V. Chandra, Tony Goldschlager, Adrian Praeger, Daniel Yu, Lee-Anne Slater

**Affiliations:** 1 Department of Neurosurgery, Monash Health, Clayton, VIC Australia; 2 Department of Surgery, School of Clinical Sciences at Monash Health, Monash University, Melbourne, VIC Australia; 3 Monash Imaging, Monash Health, Clayton, Melbourne, Australia; 4 Department of Radiology, School of Clinical Sciences at Monash Health, Monash University, Melbourne, VIC Australia

**Keywords:** Contrast, Neurotoxicity, Encephalopathy, Complication, Adverse event, Endovascular, Incidence, Risk factors

## Abstract

**Background:**

Contrast-induced neurotoxicity (CIN) is a recognised complication of endovascular procedures and has been increasingly observed in recent years. Amongst other clinical gaps, the precise incidence of CIN is unclear, particularly following intracranial interventional procedures.

**Methods:**

A retrospective study of consecutive patients undergoing elective endovascular treatment of unruptured intracranial aneurysms (UIAs) was performed. Patients with previously ruptured aneurysms were excluded. The primary aim of this study was to determine the incidence of CIN following endovascular UIA treatment. Our secondary aim was to isolate potential predictive factors for developing CIN.

**Results:**

From 2017 to 2023, a total of 158 patients underwent endovascular UIA treatment, with a median age of 64 years (IQR: 54–72), and 70.3% of female sex. Over the study period, the crude incidence of CIN was 2.5% (95% CI: 0.7 – 6.4%). The most common clinical manifestation of CIN was confusion (75%) and seizures (50%). Statistical analysis was conducted, and prolonged procedural duration was found be significantly associated with developing CIN (OR 12.55; *p* = 0.030).

**Conclusion:**

Clinicians should be aware of the risk of CIN following endovascular neurointervention, particularly following technically challenging cases resulting in prolonged procedural time.

## Introduction

With rising detection of unruptured intracranial aneurysms (UIAs), and the increasing preference for minimally invasive techniques, endovascular treatment of UIAs has become widely adopted in routine clinical practice. All intervention, however, is associated with some degree of risk, and contrast-induced neurotoxicity (CIN) has emerged as an increasingly observed complication following iodinated contrast administration. CIN presents as a spectrum of neurological symptoms including visual, sensory and motor deficits, as well as aphasia and reduced consciousness [1, 2]. CIN remains poorly understood, with a recent survey demonstrating that many clinicians believed further elucidation of CIN as clinical entity was required [3]. Although CIN is a complication of any procedure requiring iodinated contrast, in the context of neurointervention, CIN can become a clinical conundrum, due to its close resemblance to post-procedural ischaemic stroke.

The reported incidence of CIN is unclear, with some studies reporting a negligible risk of 0.15% following coronary angiography [4], and others reporting an incidence as high as 3.5% following intracranial aneurysm treatment [5]. Additionally, there is a paucity of studies reporting the incidence of CIN following intracranial interventional procedures, which are known to carry greater risk of ischaemic stroke, and consequently confound the diagnostic challenge of CIN. Furthermore, particularly in the preoperative phase of elective procedures, the risk of adverse events plays a vital role in decision making, and may have implications in patient counselling. In light of this, we conducted this study with the primary aim of investigating the incidence of CIN in the context of endovascular UIA treatment. The secondary aim of this study was to identify predictors of CIN to further aid in clinical decision making.

## Methods

### Ethical approval

This study was approved by our institutional ethics committee. All data was derived from pre-existing medical records in a deidentified manner.

### Study population

A consecutive series of patients who underwent elective endovascular treatment of UIAs between January 2017 and March 2023 were included. Patients with ruptured or previously ruptured intracranial aneurysms in any location were excluded. All cases were performed by a team of fellowship trained neurointerventionalists at Monash Medical Centre (Melbourne, Australia), a high-volume neurovascular centre servicing 1.8 million people.

### Variables and outcome measures

Demographic and clinical variables including age, sex, aneurysm location, aneurysm size, procedural technique, procedural length (defined as time from groin puncture), and comorbidities were extracted from electronic medical records. The primary outcome measure was postprocedural CIN. CIN was defined as (i) new neurological deficits occurring within 24 h post-procedurally, (ii) with urgent neuroimaging excluding ischaemic, haemorrhagic or other intracranial pathologies, (iii) biochemical exclusion of metabolic encephalopathy and (iv) clinical exclusion of other neurological conditions. Patients were evaluated by our neurology/stroke service, and neuroimaging was performed to exclude other intracranial pathologies most notably ischaemic stroke. In patients who underwent MRI, punctate diffusion restriction in a distribution not correlating to neurological symptoms did not preclude a diagnosis of CIN. Final consensus of case ascertainment was reached by the investigators, including two fellowship trained interventional neuroradiologists (LAS & RVC).

### Statistical analysis

Univariate analysis was performed to evaluate the relationship between demographic and clinical variables and the development of CIN. Fisher’s exact and Pearson Chi-square tests were employed for categorical variables. Continuous variables were analysed with Mann-Whitney test for non-parametric data. Missing data was handled using pairwise deletion. All statistical analyses were conducted with Prism 10 (GraphPad Software, San Diego, California). Statistical significance was defined as a p value ≤0.05.

## Results

### Patient population

A total of 158 procedures were performed in 158 patients with 166 intracranial aneurysms, with a median age of 64 years (IQR: 54–72), and 70.3% of female sex (Table 1). Seven patients were treated for multiple aneurysms during the same procedure. The median aneurysm size was 7 mm (IQR 5.3–10). Aneurysms were most commonly located in the Anterior Communicating Artery (ACoA) (24.7%) followed by the Ophthalmic segment of the Internal Carotid Artery (ICA) (22.9%). Hypertension was the most common comorbidity, recorded in 57.6% of patients. Thirty-three patients (20.9%) had hypercholesterolaemia, and 24 patients (15.2%) had suffered a prior stroke.

**Table 1:** Baseline characteristics

Variable		*N* (%)
***Demographics***	Female	111 (70.3)
	Age (years), median (IQR)	64 (54–72)
	Smoking status	
	Current-smoker	43 (27.2)
	Ex-smoker	49 (31.0)
	Non-smoker	66 (41.8)
***Aneurysm Characteristics***	No. of aneurysms treated	
	1	151 (95.6)
	2	6 (3.8)
	3	1 (0.6)
	Aneurysm size (mm), median (IQR)	7 (5.3–10)
	Aneurysm location	
	ICA	67 (40.4)
	Petrous	1 (0.6)
	Cavernous	18 (10.8)
	Ophthalmic	38 (22.9)
	Anterior Choroidal	3 (1.8)
	Terminus	7 (4.2)
	ACA	5 (3.0)
	MCA	6 (3.6)
	ACoA	41 (24.7)
	PCoA	11 (6.6)
	PCA	2 (1.2)
	SCA	5 (3.0)
	Basilar	27 (16.3)
	Vertebral	2 (1.2)
***Comorbidities***	Diabetes	20 (12.7)
	Hypertension	91 (57.6)
	Hypercholesterolaemia	33 (20.9)
	Prior stroke	24 (15.2)
	Prior myocardial infarction	4 (2.5)
	COPD	12 (7.6)
	Chronic kidney disease	7 (4.4)
	Prior contrast allergy	0 (0)
***Procedural Characteristics***	Length of procedure (min), median (IQR)	160 (123–195)
	Contrast volume (ml), median (IQR)	200 (135–250)
	Contrast type – Iodixanol	158 (100.0)
	Procedural Technique	
	Primary coiling	40 (25.3)
	Balloon-assisted coiling	45 (28.5)
	Stent-assisted coiling	28 (17.7)
	Flow diverter + coiling	22 (13.9)
	Flow diverter only	16 (10.1)
	Flow disruptor	6 (3.8)
	Stent + Balloon assisted coiling	1 (0.6)

### Procedural characteristics

The median length of procedure was 160 min (123–195) with a median of 200 ml (135–250) of contrast administered per procedure. A number of procedural techniques and devices were employed. Primary coiling was performed in 25.3%, balloon-assisted coiling in 28.5%, and stent-assisted coiling in 17.7%. Flow diverter with coiling was utilised in 22 patients (13.9%), and flow diverter alone was employed in 16 cases (10.1%).

### Incidence and clinical presentation of CIN

Over the 6-year study period, there were 4 accounts of CIN, with a crude incidence of 2.5% (95% CI: 0.7 – 6.4%). The median age was 72 years (IQR: 66–74.5), and two patients (50%) were female. Onset of symptoms ranged from immediately to up to ten hours post procedurally. Patients experienced multiple symptoms. Confusion was observed in three patients (75%), generalised seizures in 2 patients (50%), and visual loss, aphasia and motor weakness in one patient each (25%).

Immediate CT perfusion in all cases demonstrated no signs of acute infarction or haemorrhage. Subarachnoid and cortical contrast staining was observed in 2 patients (50%), and was differentiated from subarachnoid haemorrhage by way of dual energy CT. Two patients (50%) had significant cerebral oedema. Subsequent MRI demonstrated punctate diffusion restriction which was unrelated to the distribution of neurological symptoms, in two patients (50%). One patient developed laminar necrosis as a result of status epilepticus (SE), several days after the initial CIN onset. Another patient developed stent occlusion due to cerebral hypoperfusion from haemodynamic instability, resulting in bifrontal infarction – this event occurred 4 days following the initial CIN insult (at which point the symptoms of CIN were already resolving), and was completely distinct from the original CIN diagnosis.

Two patients required intensive care admission and intubation in the context of status epilepticus. These patients were also treated with antiepileptic medications. All four patients received intravenous fluid hydrations, and three patients were administered corticosteroids. One patient was also treated with mannitol for management of cerebral oedema.

Two patients were discharged with complete resolution of symptoms 5 days following the onset of CIN. Two patients were discharged to a rehabilitation facility with ongoing deficits. These patients were deemed to have developed deficits separate from the initial CIN insult. One patient suffered a stent occlusion which occurred several days after the initial CIN presentation. The other patient developed laminar necrosis due to poorly controlled SE.

### Predictors of CIN

Analysis of the correlation between CIN and demographic, clinical and procedural variables was conducted. Univariate analysis demonstrated an association between procedural length greater than 200 min (OR 12.55; 95% CI 1.75–164.10; *p* = 0.030) and developing CIN. There were no statistically significant relationships between CIN and other investigated variables (Table 2).

**Table 2:** Predictors of contrast-Induced neurotoxicity

Variable	Univariate		
	Odds Ratio	95% CI	p value
Age > 65 years	3.33	0.48–43.70	0.352
Female	0.41	0.03–5.89	0.634
Posterior circulation aneurysm	1.18	0.09–8.09	> 0.999
Aneurysm size > 7 mm	0.31	0.02–2.12	0.358
Primary Coiling	0.00	0.0-3.03	0.573
Contrast volume > 250 ml	3.92	0.44–57.31	0.272
Procedure length > 200 min	12.55	1.75–164.10	0.030*
Active smoking	0.89	0.07–6.10	> 0.999
Preprocedural eGFR < 45	0.00	0.00-45.77	> 0.999
Diabetes	2.37	0.17–16.46	0.421
Hypertension	U	0.73-U	0.138
Hypercholesterolaemia	0.00	0.00-3.92	0.580
Prior stroke	0.0	0.0-5.90	> 0.999
Prior myocardial infarction	0.0	0.0-40.56	> 0.999
COPD	0.0	0.0-13.84	> 0.999
Chronic kidney disease	0.0	0.0-27.77	> 0.999

## Discussion

The aim of this study was to investigate the incidence of CIN following elective endovascular UIA treatment, in order increase knowledge of CIN as a clinical entity, as well as improve decision-making and patient counselling in the preoperative planning phase. Over the 6-year study period, the incidence of CIN in the treatment of UIA was 2.5% (95% CI: 0.7 – 6.4%). Moreover, prolonged procedural time was a significant predictor of developing CIN.

**Fig. 1:**
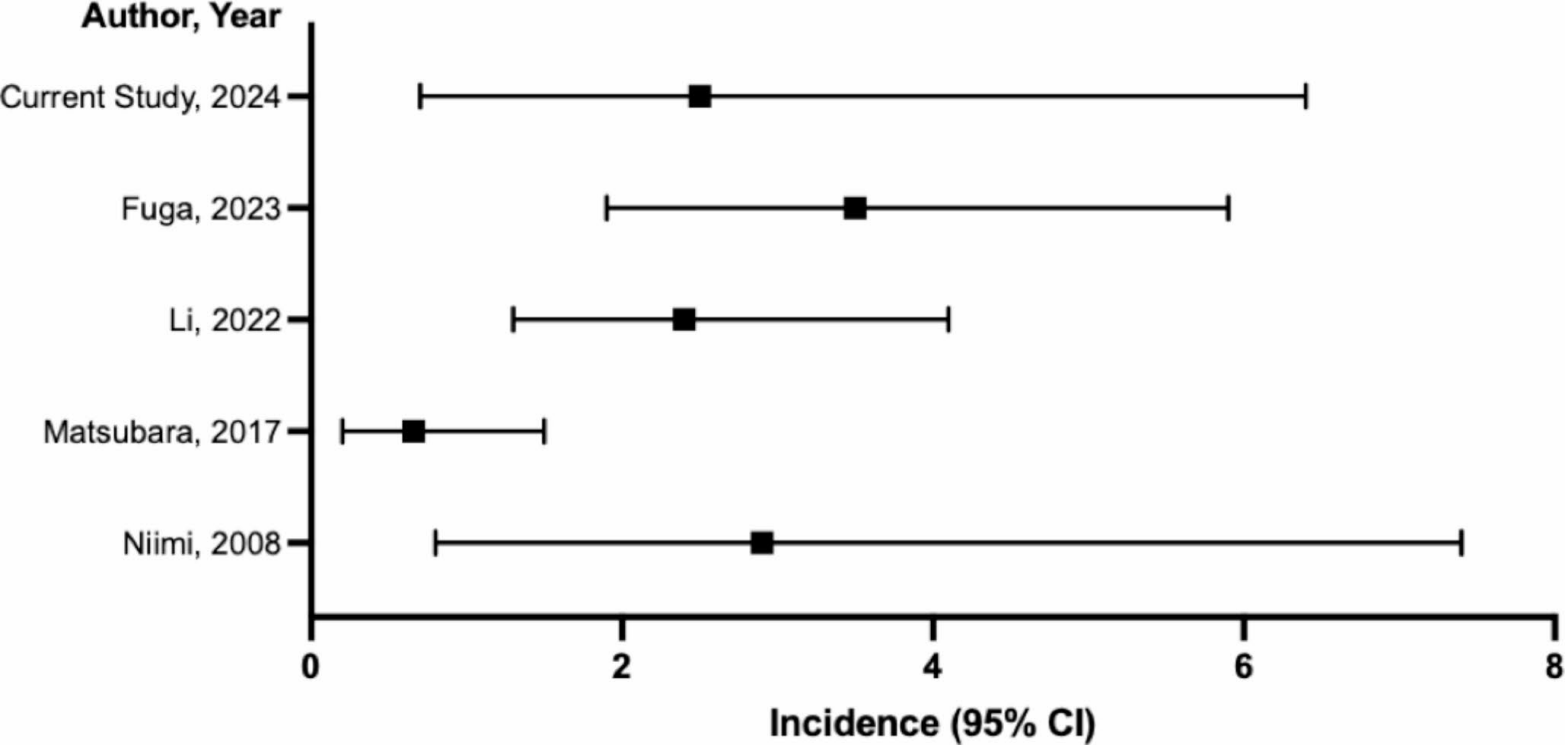
Summary of the published literature on the incidence of CIN following endovascular aneurysm treatment

The reported incidence of CIN in the literature is heterogenous. A systematic review summarising seven studies reported the incidence of CIN to be 0.51% (95% CI 0.3% – 1.0%), although this review included cohorts of coronary angiography patients and diagnostic cerebral angiography, with one study describing an incidence as low as 0.15% [4]. In the context of endovascular aneurysm treatment alone, however, the incidence ranges from 0.7 to 3.5% (Fig. 1) [5–8]. The findings of our study appear to be congruous with the reported incidence of CIN following UIA treatment. Compared to the general literature, however, the risk of CIN appears to be greater following UIA treatment when compared to other contexts such as diagnostic angiography. Compared to purely diagnostic procedures, neurointervention involves increased manipulation within the cerebral vasculature, and typically requires greater volumes of contrast administration as well as longer procedural durations, which may explain the greater observed incidence. Ultimately, the findings of this study reflect the experience of only a single neurovascular centre, and further prospective study on a larger scale would be required to validate and clarify the incidence of CIN following UIA treatment as well as other contexts.

The pathophysiology of CIN remains to be elucidated, although the blood-brain barrier (BBB) appears to play a central role in pathogenesis. Under normal circumstances, contrast media do not bypass the BBB. However, factors such as hypertension [2], prior ischemic stroke [9], and even contrast agents themselves [10], may lead to disruption of the BBB, ultimately allowing passage of contrast into the brain [11]. Subsequent to this, the manifestation of CIN has been suggested to be caused by the direct neurotoxic effects of contrast on the brain parenchyma [12], as well as cerebral oedema due to oncotic pressure changes with the outflux of hyperosmolar contrast media [1, 13]. It has been suggested that the severity of BBB disruption is proportional to the concentration of contrast and the length of time contrast exists within the cerebrovasculature [14]. In addition to the aforementioned factors surrounding UIA treatment, greater physical manipulation of the cerebrovasculature may have detrimental consequences on the BBB due to shear stress as well as physical damage on a cellular level, further explaining the observed incidence of CIN following interventional versus diagnostic procedures. Ongoing study into the pathophysiology of CIN may elucidate the implications of this relationship further.

In the present study, a prolonged procedural length was associated with developing CIN. The reasons for this are beyond the scope of this study, however this finding is very likely to be explained by the link between BBB breakdown and prolonged cerebrovascular exposure to contrast agents. It may also be that longer procedural duration is associated with more technically challenging cases, which may result in increased disruption of the BBB from increased intravascular manipulation. Preoperatively, this is an important consideration, and highlights the vital importance of a thorough preoperative clinical and radiological assessment, including evaluation of anatomical variation and aneurysmal characteristics.

In addition to prolonged procedural duration, several factors including prior stroke, renal failure and hypertension have been reported to increase the risk of CIN [2, 5, 7, 15]. Ischaemic stroke causes injury to the BBB as a result of hypoxic damage to tight junctions and the vascular endothelium [9], and may increase the likelihood of contrast passage leading to manifestation of CIN. Similarly, hypertension increases shear stress on the vascular wall degrading the integrity of the BBB [15, 16]. Renal dysfunction, on the other hand, may impair contrast clearance, leading to increased accumulation of contrast in the cerebrovasculature [7]. Consideration of these clinical factors and comorbidities may play an important role in risk stratification as well as the implementation of preventative measures in CIN.

This study has several limitations. Firstly, due to the lack of a formalised diagnostic criteria for CIN, it is possible that CIN was underdiagnosed, which may imply an incidence that is actually higher than reported. In addition, clinical and demographic variables were retrospectively extracted from electronic medical records which may affect the reliability of results. For example, the volume of contrast administered is often recorded as the total amount of contrast opened during the procedure, although this may not precisely reflect the exact amount injected. Additionally, several relevant data points were unable to be accurately retrospectively extracted, including details relating to contrast administration and procedural technique, which may have provided relevant insight. Moreover, the retrospective nature of this study also makes it susceptible to selection bias. Furthermore, the relatively low incidence of CIN may have limited the statistical power of risk factor analysis, which may have demonstrated additional predictors if the sample size were larger. Finally, restricting the study to a cohort of patients with UIAs only, may limit the generalisability of findings.

Further investigation into the incidence and risk factors of CIN is certainly warranted. The findings of this study, including the crude incidence of CIN, is based on the experience from a single neurovascular centre. Prospective studies with large sample sizes would be vital to validate the findings of our study, and further clarify the incidence of CIN following elective UIA treatment as well as other procedures such as diagnostic angiography, endovascular treatment of subarachnoid haemorrhage and mechanical thrombectomy. Further study into the risk factors associated with CIN is also imperative, which may allow for the development of preventative measures, and identification of at-risk population groups.

## Conclusion

Over the 6-year study period, the crude incidence of CIN following endovascular treatment of UIAs was 2.5% and prolonged procedure time was a risk factor for developing CIN. Clinicians should be aware of the risk of CIN in intracranial intervention, particularly in technically challenging cases anticipated to have an extended duration. Larger multicentre cohorts are required to validate the findings of this study, and to further clarify risk factors associated with CIN.

## Data Availability

No datasets were generated or analysed during the current study.
